# How Can Physical Activity Referral Rates for Breast Cancer Patients be Increased?

**DOI:** 10.3389/fonc.2016.00198

**Published:** 2016-09-12

**Authors:** Martyn Queen, Christina Karatzaferi, Saul R. Bloxham, Udaiveer Panwar, Philip Drew, Andrew G. Barton, Andrew M. Edwards, Giorgos K. Sakkas

**Affiliations:** ^1^Faculty of Sport and Health Sciences, University of St Mark and St John, Plymouth, UK; ^2^Oncology Department, Plymouth Hospitals NHS Trust, Plymouth, UK; ^3^Department of Breast Care, Royal Cornwall Hospitals NHS Trust, Truro, Cornwall, UK; ^4^Research Design Service South West, Plymouth Hospitals NHS Trust, Plymouth, UK

**Keywords:** physical activity, breast cancer, referral rate

## Introduction

Exercise therapy is beneficial for cancer survivors’ biopsychosocial aspects of health (1–3); however, the rates of exercise referrals by the oncology providers (OPs) and supporting teams remain low, causing a paradox. We chose to address this issue in this opinion article. We discuss the possible barriers that make the OPs unable or reluctant to refer more patients to exercise therapy sessions and also briefly address issues of patients’ adherence. Finally, the available exercise therapy infrastructure is discussed as an additional barrier to the therapeutic benefits of exercise. Our rationale is based on the fact that physical activity (PA) can enable wide-reaching benefits for the recovery of cancer patients during and after cancer treatment (4–6). Moreover, and specifically for breast cancer survivors, the recent trials and a systematic review disproved the notion that arm exercise should be avoided (i.e., postoperative progressive arm resistance training does not precipitate or exacerbate lymphedema) (7–9). This further supports the beneficial effects of PA across a range of contexts.

In the UK, 1.6 million cancer survivors out of a total of 2 million do not meet the minimum recommended PA guidelines for sustaining an independent and disability-free lifestyle (10). A single-blinded randomized control trial has shown that cancer survivors are more likely to become physically active if they are advised to do so by a health-care professional (11). This highlights the impact OPs have on patients’ life and survival, especially when the treatments involve a life-changing approach, such as increasing PA levels, commitment to exercise training, and adopting a healthier lifestyle.

Based on our own knowledge on cancer care practices and on research experience in other patient populations (12), we hypothesized that the main reasons behind the exercise referral paradox for breast cancer survivors in England are (1) lack of time during a consultation to promote PA, (2) OPs and supporting team lack of current knowledge and awareness of the benefits of PA in cancer survivors’ biopsychosocial aspects of health, and (3) the lack of knowledge and confidence from the patients’ side to request an exercise referral from the OPs.

## Cancer

Cancer diagnosis has a profound impact on a patient’s personal and family life (13). The ramifications of the medical interventions required to treat cancer reach beyond the physical sphere, extending to financial (14), psychological (15), and social domains (16). In the UK, approximately 338,623 new patients with cancer are diagnosed per year of which 45,000 are new patients with breast cancer, with care and support costs, beyond initial treatment predicted to reach at least £1.4 billion every year by 2020 (17). Specifically in England, the NHS costs for caring for inpatients with breast cancer have been predicted to rise to £87million by 2020 (18). This may be due to higher survival rates and increases in patients with new cancer diagnoses each year. Many patients may also need treatment for comorbidities, such as diabetes, metabolic syndrome, hypertension, and mental disorders (18), which are repeatedly linked to inactivity and disuse (19). The NICE guidelines recommend that patients with a cancer diagnosis participate in regular PA as a means of improving their prognosis and survival rates (20). However, no specific information has been provided for cancer survivors in the guidance “Exercise Referral Schemes to Promote PA” (20) nor to OPs for supporting them, while to our knowledge, cancer exercise advocacy is so far restricted to charity foundations (18) and some non-medical professional bodies, such as ACE (21). Thus, this is still far from becoming a mainstream part of cancer care pathways.

A systematic review of the determinants of exercise adherence for breast cancer survivors has shown large variations in adherence rates (22). For example, in a study from Canada participating 130 patients, the adherence was at 49% (23), while in a study from USA, the adherence rates was average 69.76% with higher rates during the first weeks (week 2 – 90.7%) (24). However, comparisons between the studies identified in the systematic review were challenging due to the differences in study design, duration, and types of exercise intervention (22). In the USA and Canada, PA levels for recovering breast cancer patients have also been reported to be as low as 37 and 27%, respectively (25, 26). In a recent survey by our team (27) in one hospital trust in the Southwest of England, we found that breast cancer diagnosis made up the 12% (*n* = 1361) of all cancers from 2013 to 2015 (3 years period). From the 1361 breast cancer patients, only 13.2% (*n* = 180) were referred into the 3 exercise referral schemes run in the city, while only 47% (*n* = 84) of those were able to adhere to the program after 6 months. For Southwest England, due to both the low referral rates and low adherence to PA programs, only the 6.2% of breast cancer patients stand to gain from the benefits of PA.

## Barriers to Exercise Referral

As mentioned earlier, our own observations show that only 13 out of 100 breast cancer survivors in the SW of England are referred to exercise. To be referred of course means that PA and exercise were discussed to start with since there is not a systematic approach for exercise referral, and therefore it depends on the random appreciation of exercise by an individual oncologist and supporting team. In an older study from Canada, approximately 60% of cancer survivors reported that exercise was not discussed with their oncologists (28). In a UK postal survey by Daley et al. (29), less than 15% of consultant breast cancer oncologists/surgeons responded (102 out of 710), of whom only 44% replied that they do discuss PA issues with their patients, with advice staying mostly “generic” (i.e., continue activities and start walking) and little mention of possible PA benefits on recurrence rates. Low rates of exercise counseling have been observed in other frailty causing chronic conditions, such as end-stage renal disease (ESRD), and were directly linked to the specialist in charge (e.g., only 38% of nephrologists were found to counsel ESRD patients to take up exercise) (12). On the other hand, it is encouraging that, in a UK online survey of 400 oncology health professionals, only 1 in 10 OPs and nurses still believe that it is more important to encourage cancer patients to “rest up” than undertaking any PA (30). However, the same survey revealed that more than half of the OPs know little or nothing about the benefits of high levels of PA in preventing or managing treatment side effects during cancer therapy, and only 6% of them are able to talk to their cancer patients about ways of increasing PA or participate in organized exercise (30). Moreover, 72% of the GPs and 60% of the OPs say almost “nothing” to their patients about the importance of PA in the management of cancer therapy side effects or the overall “after-therapy” life and survival (30). A possible explanation for these low rates of referral is that the promotion of PA is not currently part of routine cancer care nor a priority for time-pressed OPs (30). However, patients themselves would prefer if their oncologist initiated a discussion on exercise (28). As mentioned earlier, if discussion is never initiated, PA and exercise may be never addressed.

Nyrop et al. (31) highlighted that most PA communications come from oncologists (50%) compared with other clinicians interacting with the cancer patients (20%). From the 300 communications, OPs had with breast cancer patients only 35% (*n* = 105) of them resulted in aspects related to PA and promotion of an active lifestyle. Nyrop et al. (31) also found that there were no significant differences in PA communication among cancer site, patient sex, or race highlighting even further the important impact of the OPs opinion into shaping up the patients after cancer life. This suggests that there is a significant opportunity gap that OPs are missing to communicate the benefits of PA with their patients.

Time constrains appear to be the larger barrier for OPs to consult patients on PA: an Australian study examined the content of initial medical and radiation oncologist consultations (32) and found that those two types of OPs have different approaches driven by the different available consultation durations. The medical oncologist spent an average of 36 min with the patient while the radiation oncologist 23 min. The majority of the consultation from both types of oncologists focused on history and symptoms, diagnosis, prognosis, and treatment. Only 11 s were spent for checking patients’ understanding. During the consultation, the oncologists also made 37.6 informative/educational statements and asked 46.6 questions primarily about history and symptoms (32). The amount of information that an oncology consultant has to communicate to a patient during an initial consultation could explain why PA communications tend to be low or minimal. This would further support the argument that other OPs (oncology nurses or practice nurses) would be better placed to discuss the importance of PA with breast cancer patients and take responsibility for referring them into appropriate exercise programs.

Research indicates that patients would like exercise information and opportunities, to feature as part of their care pathway, yet in practice, this is variable and often dependent on clinician’s personal regard for exercise (29). In addition, whether a patient’s education programs regarding the benefits of exercise in managing their disease will make them feel confident to request from the OPs a PA referral need to be investigated. Those clinicians who favor exercise often have no suitable programs to which referrals can be made, and clinical trials are often restricted to specific cancer types. Thus, despite the known efficacy of exercise programs to support the rehabilitation of cancer survivors (33–35), the availability of community-based exercise programs is sparse.

After the OPs impact, the role of GPs is crucial in the long-term management of a patient, perhaps more so for women and older people (22) as well as in people with mental health issues (21). Qualitative research has highlighted the value of a long-term PA intervention in a Primary Care setting, through increases in PA levels and patients perceived health status (19). An Australian study on the feasibility of a governmental GP-based program (SNAP) found that GPs could play an important role in supporting lifestyle behavioral programs (including PA uptake) with GPs themselves reporting that after having received relevant training and skills they referred more frequently (20).

## A Note on Patients’ Adherence

In the Southwest of England, we have noticed an exercise adherence rate at 6 months of 47% which is not dissimilar to the rates reported in other conditions (e.g., 62% in rheumatoid arthritis, <50% when overweight/obese patients transitioned from a supervised to an unsupervised program, etc.) (36, 37). Of course, adherence is a complex multifactorial issue and is beyond the scope of this article. Interestingly, adherence rates for PA programs tend to be higher from a practice or specialist nurse referral (38) due to longer consultation periods that practice nurses have to engage patients (38). This further highlights the importance of contact time between health-care provider and patient for the overall adherence to therapy. Therefore, due to time constrains, it seems that nurse practitioners may be better placed than OPs and GPs to engage patients with PA schemes. However, the authority and the impact that the OPs have on patients’ life options should not be underestimated.

## Conclusion

It seems that the problem appears to be twofold: a lack of capacity for oncologists to discuss and direct patients to PA programs and a lack of knowledge of the impact of exercise on the recovery process for breast cancer patients. Substantial evidence exists on the benefits of PA for cancer recovery, yet referral rates for PA remain low.

More research is needed in order to find the most effective approach for improving OPs referral rates.

## Author Contributions

MQ: design, data collection, drafting, writing and editing the paper. CK: reviewing and editing the paper. SB: reviewing and editing the paper. UP: reviewing and editing the paper. PD: reviewing and editing the paper. AB: reviewing and editing the paper. AE: reviewing and editing the paper GS: idea, design, drafting, and editing the paper.

## Conflict of Interest Statement

The authors declare that the research was conducted in the absence of any commercial or financial relationships that could be construed as a potential conflict of interest.
